# The high Andes, gene flow and a stable hybrid zone shape the genetic structure of a wide-ranging South American parrot

**DOI:** 10.1186/1742-9994-8-16

**Published:** 2011-06-15

**Authors:** Juan F Masello, Petra Quillfeldt, Gopi K Munimanda, Nadine Klauke, Gernot Segelbacher, H Martin Schaefer, Mauricio Failla, Maritza Cortés, Yoshan Moodley

**Affiliations:** 1 Max Planck Institute for Ornithology, Vogelwarte Radolfzell, Radolfzell, Germany; 2 Konrad Lorenz Institute for Ethology, Department of Integrative Biology and Evolution, University of Veterinary Medicine Vienna, Austria; 3 Department of Evolutionary Biology and Animal Ecology, University of Freiburg, Germany; 4 Department of Wildlife Ecology and Management, University of Freiburg, Germany; 5 Proyecto Patagonia Noreste, Río Negro, Argentina; 6 Laboratorio de Ecología y Diversidad de Aves Marinas, Universidad Católica del Norte, Coquimbo, Chile

## Abstract

**Background:**

While the gene flow in some organisms is strongly affected by physical barriers and geographical distance, other highly mobile species are able to overcome such constraints. In southern South America, the Andes (here up to 6,900 m) may constitute a formidable barrier to dispersal. In addition, this region was affected by cycles of intercalating arid/moist periods during the Upper/Late Pleistocene and Holocene. These factors may have been crucial in driving the phylogeographic structure of the vertebrate fauna of the region. Here we test these hypotheses in the burrowing parrot *Cyanoliseus patagonus *(Aves, Psittaciformes) across its wide distributional range in Chile and Argentina.

**Results:**

Our data show a Chilean origin for this species, with a single migration event across the Andes during the Upper/Late Pleistocene, which gave rise to all extant Argentinean mitochondrial lineages. Analyses suggest a complex population structure for burrowing parrots in Argentina, which includes a hybrid zone that has remained stable for several thousand years. Within this zone, introgression by expanding haplotypes has resulted in the evolution of an intermediate phenotype. Multivariate regressions show that present day climatic variables have a strong influence on the distribution of genetic heterogeneity, accounting for almost half of the variation in the data.

**Conclusions:**

Here we show how huge barriers like the Andes and the regional environmental conditions imposed constraints on the ability of a parrot species to colonise new habitats, affecting the way in which populations diverged and thus, genetic structure. When contact between divergent populations was re-established, a stable hybrid zone was formed, functioning as a channel for genetic exchange between populations.

## Background

Current molecular genetic methods allow the understanding of the genetic structure underlying different populations of a species with previously unforeseen resolution [e.g. [1-3]]. As a result, it is possible to undertake fundamental investigations in ecology and evolution, like the study of the influence of past and current environmental conditions, together with ecological barriers, in shaping the population structure of wild animal species. These studies provide a unique opportunity to understand how species have evolved and how they are organised across landscapes [4]. The constraints that heterogeneous landscapes (e.g. barriers, resource distribution) and environmental conditions (e.g. climate) impose on the ability of animals to colonise new habitats have genetic implications affecting the structure, dynamics and persistence of populations [e.g. [5-9]]. Thus, significant genetic structuring can be expected among populations where gene flow is restricted [e.g. [5,10]].

However, when contact between divergent populations is re-established, hybrid zones can form with resultant consequences for the evolutionary trajectories of the interbreeding populations [e.g. [11]]. Two scenarios could potentially occur: if hybrid fitness is high, introgression will be widespread and hybridising populations may become panmictic over time, replacing the original populations [12]; but if interbreeding is limited in geographical range, hybridising populations may experience genetic exchange without panmixia [e.g. [13]]. Under such circumstances hybrid zones may persist over time and function as channels for genetic exchange between the populations, increasing overall levels of genetic and phenotypic diversity [e.g. [14-17]].

Although Pleistocene climate conditions played an important role in initiating major phylogeographical structuring in today's fauna [18], very little is known about their effects on southern South American terrestrial vertebrates. Phylogeographic studies in this region are scarce and have been conducted on rodents [19-21], lizards [22-24], amphibians [9,25], and a bird species [26]. Several of these studies suggested that the phylogeographic patterns observed, such as past fragmentation, range expansion, and secondary contact, could in part be understood in light of Pleistocene climate conditions. There is evidence of several cycles of more arid conditions intercalated with moist periods during the Pleistocene and Holocene of Southern South America [e.g. [27-30]], which were related to glaciation events [31], influencing the vegetation distribution [32] and the fauna depending on it.

One species that may allow hypotheses about gene flow both across a heterogeneous distribution, and into hybrid zones to be tested is the burrowing parrot (*Cyanoliseus patagonus*) (Aves, Psittaciformes). This species is distributed in a particularly heterogeneous arid to semi-arid landscape, across an extensive ~1,000,000 km^2 ^range in Chile and Argentina. A previous study [33] suggested that precipitation and temperature restrict burrowing parrot distribution in Argentina. According to this study [33], burrowing parrots are restricted to an area with median annual precipitation up to 600 mm, and annual average temperatures of no less than 8°C. However, this topic merits further research, as the study [33] was based on outdated distributional data and on the plain interpretation of maps, without detailed statistical analyses. The most dominant feature of this region is the high Andes, a mountain range that attains an altitude of up to 6,900 m and appears to separate burrowing parrot populations in Chile from those in Argentina. The predominant ecosystem on the Chilean side of this region is the 'Matorral', where vegetation is adapted to the generally dry conditions of a Mediterranean climate zone [34-36]. On the Argentinean side the semi-desert scrubland known as the 'Monte' is predominant, and this occurs from Patagonia to the North-west of Argentina [[37] and references therein]. Even though there are no extrinsic barriers to parrot dispersal in Argentina, more than 2,300 km separate the southernmost and northernmost burrowing parrot populations there. In addition, burrowing parrots breed in sandstone, limestone or earthen cliffs or "barrancas" (gorges or ravines), where they excavate nest burrows and form colonies [e.g. [37]]. These cliffs are heterogeneously located in the driest parts of the burrowing parrots range, being commonly found along permanent or temporary rivers, lakeshores, and the seacoast. Given the dryness of this environment, burrowing parrot colonies are never far from freshwater on which they are completely dependent, as they need to drink several times per day [37,38] (Figure S1). These specific requirements for nest sites, which are spread over thousands of square kilometres, and water, together with the colossal barrier of the Andes, may favour the isolation of burrowing parrot breeding sites and a complex population structure driven by genetic drift.

Due to the heterogeneity of habitats within this species' range, four burrowing parrot sub-species have been proposed, three of which are found in Argentina: *C. p. patagonus *in Patagonia, *C. p. andinus *in the Cuyo region to the west and north-west, and *C. p. conlara *ranging in the San Luis region between the former two (hereafter *patagonus, andinus, conlara*) [34,39,40]. The sub-species *C. p. bloxami *(hereafter *bloxami*) is found on the Andean foothills of Central Chile [34,41]. Three of the sub-species, namely *andinus, patagonus *and *bloxami*, are clearly morphologically distinct (size and plumage coloration) [34], while some authors [34,40,42] considered *conlara *a hybrid, owing to its intermediate geographic location and phenotype, between *patagonus *and *andinus*. Little is known about the genetic structure of burrowing parrots and how this corresponds to the morphological sub-species described above. A previous study [43] attempted to address this using seven microsatellite markers and suggested moderate differentiation between *bloxami *and all other subspecies, but differentiation within Argentinean samples was not detected. Analysis of a larger sample using a uniparental marker such a mitochondrial DNA may increase the resolution of genetic structure in this species.

Burrowing parrots are currently threatened by intense collection of birds for the pet trade [37], unjustified persecution as a crop pest [41,44] and strong habitat loss and degradation, particularly in the Monte ecosystem [45]. The latter could strongly reduce connectivity among the populations, enhancing isolation. As key species in the Monte, any negative impact on burrowing parrots could potentially affect other species since their abandoned and semi-collapsed nests provide breeding space to many other cavity nesters (such as insects, reptiles, birds and small mammals) [46].

Given the marked phenotypic differences between the populations on both sides of the Andes we hypothesise that gene flow across this high mountain range, the largest barrier in the region, must be severely restricted. We tested this hypothesis using three mtDNA loci, in a large scale sampling effort covering almost the entire species range. We also aimed to uncover the underlying population structure of this species, determine their geographic origins and suggest possible routes of colonisation. We also used these data to determine if an *andinus-patagonus *hybrid zone exists. Lastly, considering the conservation value of this key species, its potentially restricted distribution with respect to climatic factors, and the unchecked degradation of their preferred habitats, we aim to ascertain the extent to which ecological and climatic factors influence their population structure.

## Methods

### Samples

**Figure 1:**
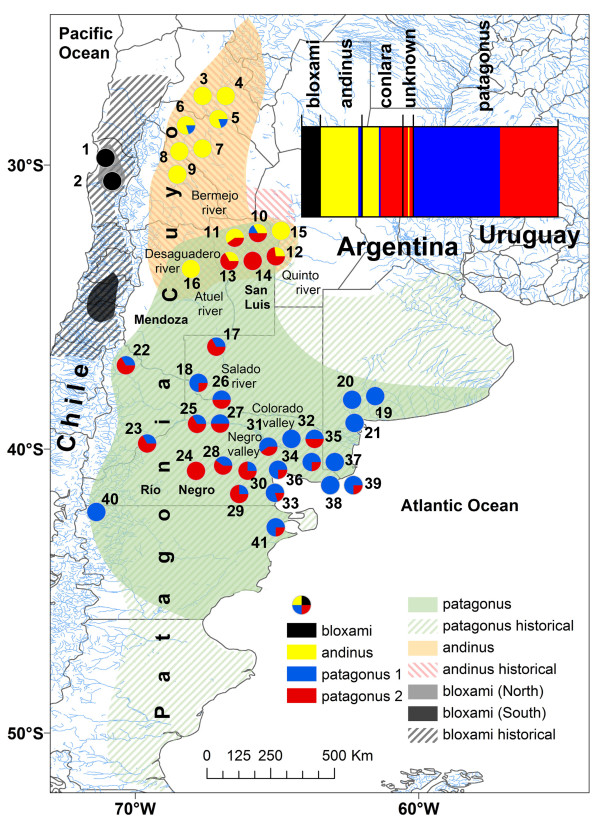
**The distribution of burrowing parrot (*Cyanoliseus patagonus*) haplotypes in Chile and Argentina**. The historical (dashed areas) and current distribution (coloured areas) of the different morphological subspecies is depicted. The proportion of haplotypes at each sampling location (see Additional file 1 Table S1 and Additional file 2 Table S2) that were assigned to different burrowing parrot populations is displayed in population pie charts. Numbers correspond with each sampling location (see Table 1). The inset shows the plot of the Bayesian assignments for all sampled individuals to an optimal four populations as determined by BAPS v5 (mixture model). The individuals are grouped taxonomically.

Recently, various aspects of the breeding biology of this species have been investigated extensively, providing the necessary framework for this study [e.g. [37,38,47-57]]. Fieldwork was carried out from November to December 2007 (Argentina), February 2008 (northern Chile) and from October to November 2008 (Argentina). Thirty-three colonies and eleven roosting places of the four previously proposed sub-species (*bloxami, andinus, conlara *and *patagonus*) were visited and naturally moulted feathers were non-invasively collected (Figure 1). Since burrowing parrots moult their primary feathers at the beginning of their breeding season, from November onwards [38,58], collection is usually straightforward, as feathers tend to accumulate at the bottom of the cliffs with colonies. Taxonomic assignment of colonies was conducted following previous studies [34,39]. The southernmost populations of *bloxami *in Chile were not accessible.

### Molecular methods

DNA was extracted from the feather quill, for a subset of 3 - 29 individuals from each of 44 populations using the DNAeasy blood and tissue kit (Qiagen, Germany). Mitochondrial genes cytochrome *b *(cyt*b*), cytochrome oxidase subunit I (COI) and ATPase subunits 6/8 were amplified via PCR using the following mitochondrial-specific primers: L15424 5'- ATCCCATTCCACCAT ACTACTC, H15767 5'- ATGAAGGGATGTTCTACTACTGGTTG-3'( cyt*b*, 586bp) [59], COI-F 5'- CCTGCAGGAGGAGGAGAYCC-3', COI-A 5'- AGTATAAGCGTCTGGGTAGTC-3' (COI, 455bp) [60], and CO2GQL 5'- GGACAATGCTCAGAAATCTGCGG-3', CO3HMH 5'- CATGGGCTGGGGTCRACTATGTG-3' (ATP6/8, 818bp) [61]. PCRs were conducted in 20µl reaction volumes containing 100 ng DNA template, 10 mM of each primer, 10 mM dNTPs (Roth, Karlsruhe), 3.125 mM MgCl, 5 U Taq Polymerase (Qiagen Taq Polymerase Core Kit) in a 1× PCR reaction buffer. PCR commenced with denaturation at 94°C for 3 minutes, followed by 35 cycles of denaturation at 95°C for 45 s, annealing at 55°C (CO1, ATP6/8) or 52°C (cyt*b*) for 45 s and extension at 72°C for 45 s. A final extension step (10 min at 72°C) concluded the PCR. Products were checked on a 1% agarose gel and purified of primers and excess dNTPs using either exonuclease-shrimp alkaline phosphatase (Fermentas Life Sciences) or the MinElu-te PCR Purification Kit (Qiagen), following the manufacturers specifications. PCR products were then sequenced in both directions by either Qiagen (Qiagen GmbH, Sample & Assay Technologies, Hilden, Germany) or by using Big Dye chemistry (Applied Biosystems) and run on an AB 3130*xl *genetic analyser (Applied Biosystems). Sequences were checked, edited and aligned in CLC DNA Workbench 5.5 (CLC bio). A total of 150 individuals from 41 of the attempted 44 locations were suitable for downstream analyses (Table 1, 2, 3 and 4; see also Additional file 1 Table S1).

**Table 1:** Summary of DNA polymorphism of burrowing parrots (*Cyanoliseus patagonus*) from Northern Chile and Catamarca, La Rioja, and San Juan in Argentina

Locality	ER	Source	**Spp**.	PS	S	A	N	H	Hd	Pi (JC)
**CHILE**										
**IV Región**										
1) Santa Gracia	MAT	C	*bloxami*	75	29	29	4	4	1.000	0.00260
2) Quebrada de San Carlos	MAT	C	*bloxami*	400	29	29	7	4	0.714	0.00123
**ARGENTINA**										
**Catamarca**										
3) Los Morteros, Abaucán river	MSB	C	*andinus*	100	15	11	4	2	0.500	0.00054
4) Salado river	MSB	C	*andinus*	90	10	5	2	2	1.000	0.00162
**La Rioja**										
5) San Blas	MSB	C	*andinus*	20	20	7	5	3	0.800	0.00237
6) affluent, Vinchina river	MSB	C	*andinus*	60	20	14	5	3	0.700	0.00216
7) Los Tambillos	MSB	C	*andinus*	10	4	4	2	2	1.000	0.00162
8) Zanja de la viuda	MSB	C	*andinus*	15	7	7	1	1	0	0
**San Juan**										
9) Huaco	MSB	C	*andinus*	20	4	4	2	2	1.000	0.00162

Eco-regions: MAT, matorral; MSB, monte de sierras y bolsones; CHS, Chaco seco; ESP, espinal; MLM, monte de llanuras y mesetas; PAM, pampa; ETP, estepa patagónica. ER: eco-region; Source, origin of the feathers used to obtain DNA: C, colony; RP, roosting place; Spp., morphological sub-species according to [34,39]; PS, estimated size of studied populations: nests in the case of colonies, individuals in roosting places; S, number of feathers collected; A, number of samples for which sequencing was attempted; N, the number of sequences; H, number of haplotypes; Hd, haplotype diversity; Pi, nucleotide diversity

**Table 2:** Summary of DNA polymorphism of burrowing parrots (*Cyanoliseus patagonus*) from San Luis, Córdoba, Mendoza, La Pampa, Buenos Aires and Neuquén in Argentina

Locality	ER	Source	**Spp**.	PS	S	A	N	H	Hd	Pi (JC)
**San Luis**										
10) San Martín stream	CHS	C	*conlara*	180	8	8	6	4	0.800	0.00360
11) Las Chacras	CHS	C	*conlara*	170	12	7	5	4	0.900	0.00421
12) Paso Grande	ESP	RP	*conlara*	1500^a^	32	15	11	6	0.727	0.00300
13) San Luis	CHS	C	Undetermined	20	8	5	3	3	1.000	0.00360
14) Quinto river	ESP	C	Undetermined	100	15	5	3	3	1.000	0.00144
**Córdoba**										
15) Piedras Blancas stream	CHS	C	*conlara*	100	11	7	2	1	0.000	0.00000
**Mendoza**										
16) Pichi Ciego	MLM	C	*andinus*	50	11	7	3	3	1.000	0.00216
**La Pampa**										
17) Algarrobo del Aguila	MLM	RP	*patagonus*	2000	15	8	6	5	0.933	0.00230
18) Colorado river	MLM	C	*patagonus*	660	52	9	4	2	0.500	0.00162
**Buenos Aires**										
19) Sierra de la Ventana	PAM	C	*patagonus*	50	21	6	4	2	0.500	0.00027
20) Tornquist	PAM	C	*patagonus*	40	1	1	1	1	0	0
21) Bahía Blanca	ESP	RP	*patagonus*	3000	29	7	3	1	0	0
**Neuquén**										
22) Tricao Malal	ETP	C	*patagonus*	Unknown	4	4	3	2	0.667	0.00180
23) Bajada Colorada	MLM	C	*patagonus*	1060	77	8	3	3	1.000	0.00180

For abbreviations see Table 1

^a ^Source [83]

**Table 3:** Summary of DNA polymorphism of burrowing parrots (*Cyanoliseus patagonus*) from Río Negro and Chubut in Argentina

Locality	ER	Source	**Spp**.	PS	S	A	N	H	Hd	Pi (JC)
**Río Negro**										
24) Los Menucos	ETP	RP	*patagonus*	300	54	8	2	2	1.000	0.00269
25) Paso Córdoba	MLM	C	*patagonus*	10	10	6	3	3	1.000	0.00287
26) Casa de Piedra	MLM	C	*patagonus*	40	22	8	4	3	0.833	0.00162
27) Villa Regina	MLM	C	*patagonus*	50	13	8	2	2	1.000	0.00323
28) Ministro Ramos Mexía	MLM	RP	*patagonus*	300	33	9	5	3	0.700	0.00183
29) El Tembrao	MLM	RP	*patagonus*	300	91	9	4	4	1.000	0.00242
30) Valcheta	MLM	RP	*patagonus*	300	34	8	4	4	1.000	0.00171
31) El Solito	MLM	C	*patagonus*	130	20	8	5	5	1.000	0.00248
32) El Saladar, Bajo del Gualicho	MLM	C	*patagonus*	5	1	1	1	1	0	0
33) Las Grutas	MLM	C	*patagonus*	420	17	6	5	3	0.700	0.00162
34) San Antonio Oeste	MLM	RP	*patagonus*	20	30	6	4	3	0.833	0.00180
35) Conesa	MLM	RP	*patagonus*	2,000	32	13	2	2	1.000	0.00323
36) raft area, Guardia Mitre	MLM	C	*patagonus*	140	25	4	4	2	0.500	0.00135
37) IDEVI	MLM	RP	*patagonus*	2,000	11	4	3	2	0.667	0.00036
38) La Lobería	MLM	C	*patagonus*	3,700	30	6	3	2	0.667	0.00108
39) El Cóndor	MLM	C	*patagonus*	37,000	49	4	4	3	0.833	0.00162
**Chubut**										
40) La Mina river	ETP	C	*patagonus*	5	5	5	2	2	1.000	0.00054
41) Puerto Madryn	MLM	C	*patagonus*	20	10	7	4	3	0.833	0.00171

For abbreviations see Table 1

**Table 4:** Summary of DNA polymorphism of burrowing parrots (*Cyanoliseus patagonus*)

		PS		N	H	Hd	Pi (JC)
						
Sub-species	Estimated total population size	C	RP				
*andinus*	2,000 nests^a^	365		24	13	0.888	0.00206
*bloxami*	5,000 - 6,000 individuals^b^	475		11	6	0.800	0.00163
*conlara* (and "undetermined")	1,700 individuals^c^	570	1,500^c^	30	12	0.798	0.00320
*patagonus*	43,330 nests^d^	43,330	10,220	85	25	0.890	0.00205

**Total sample**				150	51	0.943	0.00530

For abbreviations see Table 1.

Sources:^a ^[90] and this study,^b ^[41,85],^c ^[83],^d ^[37] and this study

### Analyses

Genetic variation was quantified as the number of haplotypes, nucleotide and haplotype diversity per population, and was determined using DnaSP v5 [62]. The degree of population structuring was ascertained using a Bayesian population assignment model that assumes no-admixture and that loci are linked BAPS 5 [63]. Unlike other clustering algorithms, this BAPS module allows the assumption of linkage between loci, thereby enabling population assignments using multilocus mtDNA data. The simulation was run ten times for K_max _values of five, ten and 20 potential populations.

Phylogenetic structuring among haplotypes was investigated by maximum likelihood (ML) in Treefinder [64,65], which first determined the best-fit substitution model for each gene partition. ML analyses was carried out assuming the HKY+G (cyt*b*), HKY+I (CO1) and J2+G (CO2/3) models, each optimising rate, frequency and heterogeneity parameters directly from the data. Models including a rate-heterogeneity parameter (+G) assumed five gamma categories. The significance of nodal bi-partitions was determined by 1,000 bootstrap replicates, from which an 80% majority rule consensus tree was constructed. We also constructed a median-joining network using Network 4.5.1.6 [66] for a graphical representation of the unrooted relationships and frequency of haplotypes.

Demographic parameters based on the coalescent can be useful in testing hypotheses of population history. In particular, statistics based on the distribution of pair-wise differences (mismatch distribution) [67] between individuals in a population as well as the detection of selection among selectively neutral loci may be used as signatures of past population expansion events. We used Arlequin 3.5 [68] to calculate the mismatch distributions and Fu's Fs for all populations identified by the clustering and phylogenetic methods above.

We roughly dated each node using a fossil calibration technique that incorporates rate smoothing for lineages with unequal rates of evolution. By constraining the tree topology to that of the 80% majority rule consensus tree calculated previously, we generated a range of node height estimates by running a bootstrap analyses 1,000 times in Treefinder, using the original starting parameters. The variance in node height was then taken as the spread of this variable in our data set. The fossil record for *C. patagonus *is extremely scarce, with three known fossils dated to 126 kyr [69-72]. A much earlier fossil that can also be attributed to the genus *Cyanoliseus *dates back to 750 kya [70-73]. However, since the nearest outgroup taxon for which molecular data were available belonged to the genus *Diopsittaca*, it was not possible to utilise the older fossil date in our calibration. We therefore assumed a minimum or latest date of 126 kyr for the coalescence of all *C. patagonus *lineages. We then performed the calibration analysis in Treefinder and, due to the availability of a single calibration date, we used local rate minimum deformation rate-smoothing, to account for the possibility of differing rates of lineage evolution within the phylogeny. 95% confidence intervals were generated from the spread of node heights.

We investigated the possibility of differing mutation rates among the three gene partitions in order to independently date each node using available mutation rates. However, since reliable mutation rates are currently only available for the avian cytochrome *b *gene [74], we partitioned the sequence data into cyt *b *and CO1+CO2/3 and performed identical analyses in BEAST 1.5.0 [75], using the parameter-rich GTR+G+I model (with 5 gamma categories) to check if cyt *b *mutation rates may be applied to other mitochondrial data. Plotting the resulting cyt *b *node heights against those of CO1+CO2/3 returned a correlation co-efficient (R^2^) value of 0.5858 (p < 0.001, data not shown), suggesting significantly differing rates of evolution between the two gene partitions. As a rate-smoothing option is unavailable in BEAST, we proceeded with only the cytochrome *b *data, using both the average avian mutation rate (*µ*) of 2.1%/million years (myr) and a Psittaciform-specific rate of 3.4%/myr [74].

Given the possibility of a relatively recent divergence and the apparent reliance of burrowing parrots on their habitat and associated climate [33,37], we also investigated the extent to which taxonomic, ecological and climatic factors influenced the distribution of genetic heterogeneity in the data. We fitted a linear model to our data using DISTLM 5 [76], thereby testing the influence of 32 variables (4 taxonomic, 7 ecological and 21 climatic; Table 5 and Table S1) using multiple matrix regressions. The climatic variables were selected taking into account the possible restriction of burrowing parrots distribution to certain temperature and precipitation ranges as suggested in a previous study [33]. The 21 temperature- and precipitation-related climatic variables were obtained, for each of the burrowing parrot colonies, with DIVA-GIS 7.1.7.2. [http://www.diva-gis.org], which are based on the WorldClim database [http://www.worldclim.org], version 1.3, at 2.5 minutes resolution [77]. We first assessed the marginal genetic variation explained by each set of variables separately. Since some of these variables are likely to co-vary with geography (latitude and longitude of sample locations), we controlled for this influence by also reporting the conditional residual variation remaining after the influence of geography was subtracted. Still controlling for geography, we then tested all variables together using a forward selection approach [78], which sequentially determines the variables that explained the majority of the marginal variation in the data. The significance of all DISTLM regressions was determined by 9,999 permutations.

**Table 5:** Results of the multivariate multiple regressions

Predictor Variable	Proportion of the explained genetic variation		
	Marginal	Conditional	Sequential
Phenotype (sub-species definition)	0.2204	0.0156	0.0010
Ecoregions	0.0081	0.0075	0.0010
Climate (all variables)	0.8529	0.4812	0.4812

Climate (per variable)			
Temperature Seasonality (SD * 100)	0.4856	0.2797	0.2797
Precipitation Seasonality (CV)	0.4118	0.1427	0.1100
Mean Monthly Temperature Range	0.1216	0.0472	0.0207
Precipitation of Wettest Quarter (mm)	0.0379	0.0249	0.0197
Minimum Temperature of Coldest Month	0.0195	0.0012	0.0154
Temperature Annual Range	0.3210	0.1919	0.0130
Isothermality (2/7) (* 100)	0.4364	0.2165	0.0106
Precipitation of Driest Quarter (mm)	0.2885	0.0685	0.0079
Mean Temperature of Wettest Quarter	0.1566	0.0846	0.0028
Precipitation of Coldest Quarter (mm)	0.0463	0.0080	0.0012
Precipitation of Warmest Quarter (mm)	0.0786	0.0552	--
Mean monthly minimum Temperature	0.0734	0.0605	--
Mean Temperature of Coldest Quarter	0.0510	0.0089	--
Mean Temperature of Warmest Quarter	0.2424	0.1662	--
Mean Temperature of Driest Quarter	0.0634	0.0461	--
Annual Mean Temperature	0.1296	0.0894	--
Precipitation of Wettest Month (mm)	0.0312	0.0207	--
Annual Precipitation	0.0830	0.0480	--
Mean monthly maximum Temperature	0.1615	0.0988	--
Precipitation of Driest Month (mm)	0.2880	0.0743	--
Maximum Temperature of Warmest Month	0.2987	0.1965	--

Analyses were carried out for 150 individual mitochondrial sequences of burrowing parrots, 21 bio-climatic parameters, morphological subspecies identity, and Chilean and Argentinean eco-regions.

--, added < 0.000001 to the regression sum of squares; CV: coefficient of variation; SD: standard deviation. No. of permutations = 9999. Temperature Seasonality: the standard deviation of the weekly mean temperatures expressed as a percentage of the mean of those temperatures (i.e. the annual mean). Precipitation Seasonality: the standard deviation of the weekly precipitation estimates expressed as a percentage of the mean of those estimates (i.e. the annual mean). See also Additional file 1 Table S1.

To explore the influence of the geographic landscape in more detail, we used a spatial clustering algorithm, implemented in Tess 2.3.1 [79]. This method performs Bayesian clustering given a set of input sampling locations. The program constructed Dirichlet cells around each sampling location to produce a Voronoi tessalation. Multilocus DNA sequences were then used to statistically infer population structure based on this Voronoi neighbourhood system using a hidden Markov random field prior. Parameter space was explored by Markov chain simulations. We ran the program ten times again with a K_max _of five, ten and 20, assuming no-admixture at 50,000 sweeps each, with a burn-in of 10,000 sweeps. We also included 23 dummy points, well outside the extant distribution *C. patagonus*, in order to restrict the simulation to within the species range. The resulting hard-clustering image represents the geographical clustering of individuals in landscape space, given their sampling origins and their multilocus genotypes. To allow the optimal tessalation to be viewed in geographic space, the hard clustering image with the lowest deviance information criterion (DIC) score was imported into ArcGIS 9.3, and superimposed onto a map of the region.

## Results

### Genetic variation

Complete sequences for all three mitochondrial fragments were obtained for 150 individuals from 41 locations (Tables 1, 2, 3 &4, and Additional file 1 Table S1), representing 7 ecoregions and encompassing the entire species range. However, since the quality of DNA isolated from shed feathers was low, this number was less than half of the 327 individuals from which DNA was extracted. Genetic variation was also low, with only 81 polymorphic sites from a concatenated sequence of 1,859 bp, resulting in 51 unique haplotypes (Tables 1, 2, 3 &4, and Additional file 2 Table S2). This was reflected in a species-wide haplotype diversity of 0.943 and nucleotide diversity of 0.00530 (Table 4), and given varying locality samples sizes these values ranged from 0.500 - 1.000 and from 0 - 0.0360 respectively (Tables 1, 2, &3). Considering taxonomic designations, *conlara *was found to have the highest nucleotide diversity. Non-*conlara *sampling localities, contained similar levels of diversity, with the exception of eight localities with a nucleotide diversity of less than 0.001. This is reduced to only four localities, if we exclude those with fewer than 3 individuals.

### Genetic structure

Despite low diversity, Bayesian clustering consistently structured the entire sample into four population clusters regardless of the K_max _prior, and the distributions of these are depicted in Figure 1. One of the four populations corresponded exactly to the *bloxami *phenotype, and was found exclusively on the Chilean side of the species range (inset, Figure 1). Within Argentina, only two members of the northern *andinus *phenotype did not fall within an Andinus population cluster, and the *patagonus *phenotype was divided into two populations, hereafter called Patagonus1 and Patagonus2. There was no support for the existence of the intermediate *conlara*, as this phenotype clustered either within the Andinus or Patagonus2 populations, with a single *conlara *clustered within Patagonus1.

**Figure 2:**
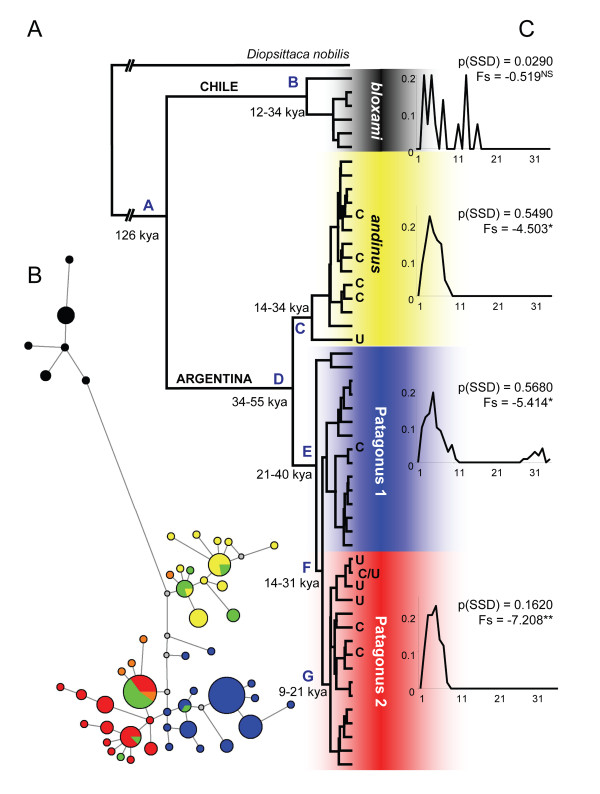
**Phylogenetic relationships and population history among burrowing parrot (*Cyanoliseus patagonus*) populations**. **A**. 80% majority-rule maximum likelihood phylogeny of 51 mitochondrial haplotypes. Nodal dates were determined assuming the coalescence of all lineages by at least 126 kya. The phylogeny was rooted by a *Diopsittaca nobilis *individual. **B**. A median-joining network showing the unrooted relatedness between haplotypes. Distances are proportional to the number of mutational changes and the size of each circle is proportional to haplotype frequency. The smallest grey circles denote unsampled haplotypes invoked by the median-joining algorithm. **C**. Mismatch distributions, the probability of rejecting the null hypothesis of a sudden population expansion p(SSD) and Fu's Fs statistic for all four populations. All clades and haplotypes are colour-coded according to Figure 1. Haplotypes belonging to the morphological sub-species *conlara *according to [34,39] are marked with "C" in panel A and are coloured green in panel B. Haplotypes where the phenotype could be assigned to neither *andinus *nor *patagonus *with certainty are denoted with "U" in panel A and are orange in panel B. Node references are in blue alphabet.

Maximum likelihood analysis recovered a fully resolved haplotype phylogeny (Figure 2A) again showing four population groups, but with Bloxami as basal and distinct from all other populations. Within Argentina, the northern Andinus forms a sister relationship with the entire Patagonus population, with the two most ancestral haplotypes of the latter sampled in the Cuyo region (locations 3 to 9, and 16, Figure 1), among phenotypically *andinus *populations. In Patagonia, the *patagonus *subspecies is divided into the genetically distinct populations Patagonus1 and Patagonus2, yet without distinguishing phenotypic characteristics. A median-joining haplotype network (Figure 2B) confirmed the large divergence separating populations on either side of the Andes, but also demonstrated that all three Argentinean populations contain star-shaped haplotype clusters in which several less-frequent haplotypes are very closely related to a single common haplotype. Both phylogeny and network showed that individuals identified as *conlara *or "undetermined" (where phenotype could not be assigned to *andinus *or *patagonus *with certainty) belonged to either the Andinus (11/33 individuals; 5/14 haplotypes) or Patagonus (Patagonus1: 1/55 individuals, 1/15 haplotypes; Patagonus2: 18/51 individuals, 6/16 haplotypes) populations (Figure 2 A/B), confirming a hybrid origin for this phenotype. The six undetermined individuals (5 haplotypes) that were sampled within the *conlara *range from a population near the town of San Luis and from the Quinto river were most closely related to confirmed *conlara *haplotypes on the median-joining network (Figure 2B), showing that both populations are admixed and therefore belong within *conlara*.

### Population History

Mismatch distributions of pair-wise nucleotide differences (Figure 2C) showed largely unimodal distributions among Argentinean populations, but was multimodal within the Chilean Bloxami. Bloxami was also the only population where the hypothesis of a sudden population expansion was rejected (p(SSD) = 0.0290). Furthermore, negative and significant Fu's Fs values, also indicating population expansion, were recovered for Andinus, Patagonus1 and Patagonus2, with the slightly negative value for Bloxami not significant. Assuming a fossil calibration of at least 126 kyr for the coalescence of all *C. patagonus *lineages, we estimated dates for all population nodes to between 9 and 55 kya (Additional file 3 Table S3). Applying the average avian mutation rate (*µ *= 2.1%/myr) to the cytochrome *b *data returned higher nodal divergence estimates with much larger 95% confidence intervals. However, the psittaciform-specific mutation rate (*µ *= 3.4%/myr) yielded estimates very similar to those of the fossil calibration method. Owing to the larger variance of BEAST estimates, the 95% interval of almost all fossil calibration dates was contained entirely within the psittaciform-rate interval (Additional file 3 Table S3). The only exception being node E, although both fossil and *µ *estimates did overlap. Indeed, if a divergence time (*T*) of 126 kyr for all burrowing parrots was substituted into the equation *µ = *node height/2*T*, a mutation rate of 3.5%/myr would result, implying that the cytochrome *b *gene evolves more rapidly among psittaciform species. The 95% confidence intervals of the fossil calibrated population nodes reflect the stochasticity in the maximum likelihood estimation and the lack of internal calibration nodes. Their accuracy, therefore, relies heavily on that of the 126 kyr fossil estimate, for which there exist three independently dated fossils [69-72]. In contrast, the much larger variance of BEAST estimates may reflect the limitations of using only a third of the total available data. We therefore continue with the more accurate fossil calibrated estimates (Figure 2A), and we view these as the latest range of dates by which each lineage splitting event could have occurred.

### Influences on genetic variation

Since extant burrowing parrot populations appear to have evolved relatively recently, one might expect to find genetic structure to be associated to present-day ecological and environmental variables. The amount of marginal variation explained by predictor variables was highest for the suite of 21 climatic variables (85%) followed by phenotype (22%) and lastly ecoregions (0.81%). This pattern held true even when the variation attributable to geography was removed in conditional tests (Table 5). When each climatic variable was tested individually, temperature seasonality, isothermality, temperature annual range, mean temperature of warmest quarter and maximum temperature of warmest month could all account for more than 15% of the conditional variation in the data set. When autocorrelations between these variables were taken into account in sequential tests, only temperature seasonality (28%) and precipitation seasonality (11%) were found to explain more than 10% of the genetic variation (Table 5).

**Figure 3:**
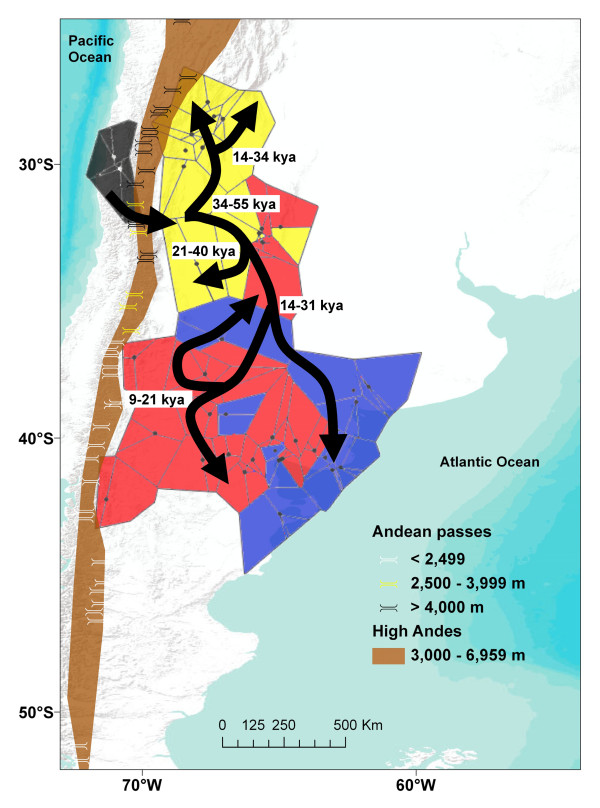
**Spatial clustering of multilocus burrowing parrot (*Cyanoliseus patagonus*) genotypes in landscape space**. The most likely hard clustering posterior was superimposed on to a map of the region to convey patterns of spatial clustering of four populations in Chile and Argentina. A model for the colonisation and diversification events that have shaped mitochondrial population structure is also depicted. Each dot represents a sampling location (see also Additional file 1 Table S1). The arrows indicate the migration model proposed for the species. A graphical representation of the high Andes in the region has been added (in light brown) together with the exact location of all known passes in the region.

The 44% of genetic variation explained by geography was partitioned into the four already-defined population clusters by TESS software. The optimal tessellation cluster was obtained consistently for all runs and for all starting K_max _values and this partitioned Chilean from Argentinean populations, as have previous analyses (Figure 2). Spatial structuring within Argentina describes genetically Andinus haplotypes in the Cuyo region, to the north of the species range, whereas in Patagonia, Patagonus1 inhabits the entire Atlantic coast and a thin wedge between southern limit of Andinus and the north of the Río Colorado valley (Figure 3). The Patagonus2 population occupies a non-contiguous area along the entire course of the Río Colorado and except for a population in which Patagonus1 is particularly dominant, also the entire course of the Río Negro until just before it enters the Atlantic Ocean. Some Patagonus2 haplotypes also range north into the San Luis region, which is the southern-eastern end of the Andinus distribution, where hybridization with Andinus results in the *conlara phenotype*.

## Discussion

### Origins and gene flow across the Andes

Although we were not able to access populations to the south of the Bloxami range in Chile, it is nevertheless apparent that very limited gene flow across the Andes has rendered the Bloxami populations both genetically and phenotypically distinct from all other burrowing parrots. This further corroborates a previous study using microsatellite loci that showed a clear separation between Chilean and Argentinean populations, but no structure within Argentina [43]. By contrast, cluster analyses of 3 mitochondrial fragments suggest a complex population structure for burrowing parrots in Argentina. Since the Bloxami split is at the root of the species phylogeny, and that this is the only population to not bear any of the signatures of a recent population expansion (Figure 2B/C), we suggest a Chilean origin for this species, with a single migration event across the Andes that gave rise to all extant Argentinean mitochondrial lineages (Figure 3). Although the sampled Chilean lineages coalesce to only 12 - 34 kya, their southern range was not sampled and the Bloxami mismatch distribution (Figure 2C) suggests a population with no significant changes in effective population size over time. Furthermore, the network of Bloxami lineages (black circles, Figure 2B) describes a population with several haplotypes in low frequency, none of which are related to each other by fewer than two mutational steps, implying that intermediate and other more distant haplotypes, that could have increased the Bloxami coalescence time, were either lost or not sampled.

In contrast, the histories of all populations in Argentina are characterised by recent (between 9 - 55 kya) expansion (Figure 2 B/C). The migration event across the Andes therefore dates roughly to between 55 - 126 kya, to a time when the southern and central reaches of that mountain range were heavily glaciated [e.g. [28,31,32,80]], making a crossing over the lower (< 2,500 m) passes in these areas impossible for want of water and shelter until at least 14 kya [80]. Instead, we suggest a crossing over one of the few intermediate (2,500 - 4,000 m) mountain passes to the north of the present-day species distribution (Figure 3). Although rare, recent evidence of exactly such a crossing from Chile into the Mendoza region of Argentina by a Peruvian pelican (*Pelecanus thagus*), where all passes are higher than 2,500 m, shows that bird migration across the high Andes is biologically possible [81]. The resulting founder effect of an Andean crossing may also help to explain the contrasting size and plumage differences that exist between *bloxami *and *andinus *[34].

### Gene and phenotype divergence in Argentina

The initial divergence of Andinus-Patagonus mitochondrial lineages during the late Pleistocene 34 - 55 kya represents a clear asynchrony between genetic and phenotypic divergence times within this species. The two most ancestral haplotypes assigned to the Patagonus1 population (Figure 2A/B) are phenotypically *andinus *individuals, sampled among *andinus *locations in the Cuyo region, at least 500 km from the *andinus-patagonus *hybrid zone in San Luis. Since older Patagonus haplotypes were not detected, despite a sample of 106 *patagonus *individuals from across their entire distribution, we propose that phenotypic divergence between Andinus and Patagonus lagged genetic divergence, by 3 - 41 kyr, which is the minimum-maximum time difference between the initial Andinus*-*Patagonus and the Patagonus1-Patagonus2 divergences. Further evidence that phenotypic differences take longer to evolve in this species is the genetic diversification of Patagonus1 from Patagonus2 within Patagonia, which occurred no later than 9 kya, yet the two populations still remain phenotypically indistinguishable from each other. This lag in phenotypic divergence may result from greater lineage sorting among mitochondrial genomes, and whether it holds true for slower evolving unlinked regions such as nuclear introns remains to be investigated.

### Colonisation and diversification

Summarising the results and available information, we propose a model for the colonisation and diversification events to have shaped the distribution of genetic variation in burrowing parrots (Figure 3). The presence of ancestral Patagonus haplotypes within the range of *andinus *implies that genetic diversification of these two populations occurred in the Cuyo region, with phenotypic diversification occurring later (after 21 kya, see Figure 2A) as Patagonus expanded south-east (see model, Figure 3). Since burrowing parrots are completely dependent on water and suitable strata in which to form nests [e.g. [37]], we predicted the population structure to be a product of isolation and hence local genetic drift. Our results suggest, rather, a low degree of isolation since most sampling localities are similarly diverse, indicating gene flow between them. This is most likely owing to suitable habitats being continuously distributed along river courses and because burrowing parrots are able to make one to four foraging trips to a distance of up to 66 km from their colonies in a single day [37]. Hence, we conclude that the south-eastern expansion of genetically Patagonus individuals probably followed the courses of the Bermejo-Desaguadero-Atuel, Colorado and Negro river systems, which were already in existence by that time [28,82]. By 14 - 31 kya, Patagonus was established along river courses, and along the Atlantic coast of Patagonia (Figure 3). The end of the last glacial maximum (LGM) ~14 kya saw the establishment of wetter, more suitable habitats in north-western Patagonia [e.g. [27-30,80]] and possibly promoted further diversification into Patagonus2 between 9 - 21 kya. Spatial clustering shows that the Holocene expansion of Patagonus2 lineages has rendered this population dominant in much of north-western Patagonia, between the Andes and the Atlantic (Figure 3), especially along and to the south of the Río Negro, where only few populations contain Patagonus1 haplotypes in high frequency.

### Secondary contact

Our results convincingly demonstrate the existence of a hybrid zone in the San Luis region, where Patagonus (Patagonus2 in all but one case) haplotypes expanding into the south-eastern range of Andinus resulted in the evolution of an intermediate phenotype: *conlara*, and this is also the most genetically diverse of the four burrowing parrot taxonomic groupings. The dynamics of hybrid zones are of great interest, especially their potential to give rise to new species or populations [e.g. [11]]. While our results warrant a thorough study of the Andinus-Patagonus hybrid zone using nuclear DNA markers, mitochondrial data reveal several processes of interest. We found no significant difference between the proportion of *conlara *individuals belonging to Andinus and Patagonus2 (*χ*^2 ^= 0.003, *df *= 1, *P *= 0.960) and Andinus and all Patagonus (*χ*^2 ^= 2.7, *df *= 1, *P *= 0.102) suggesting, firstly that there is no sex bias in dispersing Patagonus individuals, and secondly that there was no bias by resident Andinus against the choice of invading Patagonus individuals as potential mates. From our phylogeny (Figure 2A), we also conclude that the observed level of introgression resulted from at least four, possibly independent, migration events by Patagonus individuals. Within the Patagonus2 clade, four *conlara*-undetermined haplotypes comprise an entire sub-clade, suggesting that they resulted from a single successful migration event, and given the depth of this sub-clade, Patagonus2 introgression may date back to at least the latter half of the Holocene (inference, Figure 2A). Similarly, the most ancestral of Andinus haplotypes was carried by a *conlara *individual and dates back to the coalescence of the Andinus clade (14 - 34 kya). Together with the morphological conformity of the *conlara *phenotype within its range [83], this result strongly suggests a hybrid zone that has remained stable for several thousand years. The inability of seven polymorphic microsatellite loci to discriminate among Argentinean populations [43] also suggests a hybrid zone that has remained stable long enough for homoplasy to mask phylogeographic signal in hypervariable markers. Since phenotypic variation across the *andinus-conlara-patagonus *continuum, appears to be discretely, rather than continuously partitioned, implying that gene flow out of the hybrid zone is lower that gene flow into it, the possibility of the *conlara *phenotype being selectively advantageous in the San Luis region cannot be ruled out.

### The effect of climate

Our data show that up to 48% of the genetic variation can be accounted for by present-day climatic variables, considerably greater than the variation accounted for phenotype and ecoregions. This is even more surprising since ecoregions would normally be expected to co-vary with climate. This lack of variation attributable to ecoregion might be explained by the fact that the two major constraints on burrowing parrots habitat suitability, namely availability of water and cliffs, are not included in ecoregion definitions. Even the significant correlation with present-day climate appears counterintuitive since climatic conditions in the past are more likely to have had an influence over population structure. However, if we consider that the majority of the haplotypes within Patagonus1/2 and Andinus most likely evolved after the LGM 14 kya or more recently (Figure 2A), and that local climatic conditions have remained relatively similar in the last 8 kyr [e.g. [28-30,80]], this result is less surprising. Conservation implications of this are particularly important in the present-day reality of climate change.

### Conservation implications

The lack of gene flow makes the high Andes an important barrier to migration in burrowing parrots, and possibly in other bird species [e.g. [81]]. This also renders the isolated Bloxami population genetically and phenotypically distinct. This evolutionary significance is important from a conservation and management perspective. Burrowing parrots are listed as ''threatened'' species in the vertebrate red list of Chile and as such are legally protected [84]. This is because only 5,000 - 6,000 *bloxami *are distributed in the IV and VII regions of the country [41,85]. The size and uniqueness of this population means that further reductions should be avoided.

The situation differs considerably in Argentina, where burrowing parrots are officially considered an abundant agricultural pest (National Law of Defence of Agricultural Production 6704/63), despite agricultural damage occurring only locally [40,86], with very little actual crop damage recorded [37,44]. Owing to its persecution as a pest species [[37] and references therein], several colonies have been destroyed or severely reduced in size, including the extirpation of the largest known colony of some 50,000 nests [87]. Collection of burrowing parrots for the pet trade was also encouraged [88] and population reductions continued to reach levels considerable enough for the regional government of the Río Negro province to ban all hunting and trade (resolutions 23-DF-2004, 24-DF-2004, Dirección de Fauna de la Provincia de Río Negro, Argentina), thereby extending legal protection to all but seven Patagonian colonies. This protection effectively includes the bulk of the Patagonus population, approximately 40,000 nests, where genetic diversity is partitioned into two genetically distinct, yet phenotypically indistinguishable populations, which are impossible to manage separately. It should be noted, however, that 37,000 of these nests are located in a single colony - El Cóndor (sampling location 39, Figure 1) [37], which is located in an area undergoing habitat degradation that is estimated to be ten times higher (3.7% annually) [45] than the world average of 0.4% [89]. The continued existence of the burrowing parrot in Patagonia is therefore uncertain.

A negative side effect of the recent protection of Patagonian populations is the noticeable increase in commercial value of Andinus populations in the Cuyo region. All available data from this study (Tables 1, 2 &3) and the literature [90], together with unpublished data of 1 colony for which we did not obtain sequences, La Manga stream, La Rioja, 290 nests) show that the total Andinus population numbers no more than 2,000 nests. In contrast to Patagonus, where individuals are genetically but not phenotypically distinguishable, Andinus populations are distinctive both genetically (Figures 2 and 3) and phenotypically [34] from Patagonus, comprising an evolutionary significant unit that appears to be kept isolated by the Andes to the West and a stable hybrid zone to the South-east. We suggest a complete stop of trade in the Cuyo region and the development of conservation measures, particularly of the cliffs with colonies, which are crucial for the survival this population.

Most alterations in the environment, like climate change, are potential sources of new or intensified directional selection on traits important for the fitness of the species living in it [e.g. [91]]. Evolutionary responses take place on a time scale comparable to that of changes in climate, but the degree of adaptation will depend on the interplay of natural selection with processes such as gene flow, genetic drift, mutation and demography [e.g. [92]]. The tight link between genetic variation and climatic variables here reported, in light of the present-day reality of climate change, could lead to important conservation implications in burrowing parrots. Climate change is likely to impose selection pressures on traits important for fitness [e.g. [91]], thus affecting the chances of persistence of this species. Furthermore, climate change could differently affect the four population clusters detected throughout the species range and, in addition, the populations could vary in the rate of adaptation. Consequently the outcome of climate change, on top of other environmental constraints (e.g. presence of water and Monte vegetation, occurrence of cliffs), could be particularly important for some of the populations, in particular the currently endangered Bloxami and Andinus populations. Under added selection pressure, such as that imposed by ongoing climate change, populations can respond in roughly three ways: 1) by shifting in abundance and distribution, 2) by going extinct, or 3) by evolving [93]. Even if predicting which one, or even which combinations of them, will occur is difficult [92,93], some likely scenarios can be expected for the burrowing parrot population clusters. Shifts in the distribution could be possible in the case of Bloxami, distributed in Chile. Due to intensive poaching, several cliffs along the historical distribution of the species (Figure 1), which traditional contained colonies, have been found to be empty at present [41,85] (JFM pers. observ.). Provided that water and natural food items are still available in those places, and that the current small size of Bloxami is not further reduced, these colonies could be reoccupied by burrowing parrots. The situation appears to be quite different for the size-reduced Andinus population in the Cuyo region of Argentina (Additional file 4 Figure S1). In this area, only few suitable breeding places (high cliffs close to water and Monte vegetation) are available, leaving this population only two possibilities in case of strong or too sudden climate change: going extinct or evolving. For Patagonus1 and Patagonus2, two genetically distinct, yet phenotypically indistinguishable populations, the situation appears to differ again. In a few occasions, individuals from several colonies of these populations were found to breed in nests dug in human-build structures like shafts of mines, abandoned adobe buildings and wells for collecting water for cattle [94]. Additionally, some suitable breeding places are available in the southernmost as well in the easternmost areas of the historical distribution of the species. Provided the future occurrence of a more benign climate in the South and a release of human-induced pressure in the East, some of those areas could be used by burrowing parrots. However, the currently rapid habitat degradation in the region inhabited by Patagonus1 and Patagonus2 (see above) makes this possibility very uncertain, as in fragmented landscapes, rapid climate change has the potential to overwhelm the capacity for adaptation of the populations and dramatically alter their genetic composition [95]. Altogether, how closely adaptation can be expected to accommodate climate change and the additional pressure of habitat loss and fragmentation, remains a question for further research.

## Competing interests

The authors declare that they have no competing interests.

## Authors' contributions

JFM and PQ conceived and designed the study. YM, GS and MS planned the genetic analyses. JFM, PQ, MF and MC carried out the extensive fieldwork. GKM, NK and JFM generated the molecular data. JFM, NK and YM participated in bioinformatic analyses. YM, JFM and PQ were responsible for data analysis and drafted the manuscript. GS and MS reviewed the final draft of the manuscript. All authors read and approved the final manuscript.

## Supplementary Material

Additional file 1 **Table S1**. Classification, GenBank accession numbers, and sample locations with detailed climate parameters of 150 burrowing parrots (*Cyanoliseus patagonus*). Click here for file

Additional file 2 **Table S2**. Haplotype distribution of 150 burrowing parrots (*Cyanoliseus patagonus*). Click here for file

Additional file 3 **Table S3**. Divergence estimates using fossil calibration with rate smoothing across three gene partitions in comparison with cytochrome *b *mutation rates. Click here for file

Additional file 4 **Figure S1**. Sample locations (black dots), main places, regions, and ecoregions mentioned in the text, and rivers of Southern South America. Click here for file
