# Supplementary figures and images for: The high Andes, gene flow and a stable hybrid zone shape the genetic structure of a wide-ranging South American parrot
Source: Front Zool. 2011 Jun 15;8:16. doi: 10.1186/1742-9994-8-16 (PMC3142489; doi:10.1186/1742-9994-8-16)

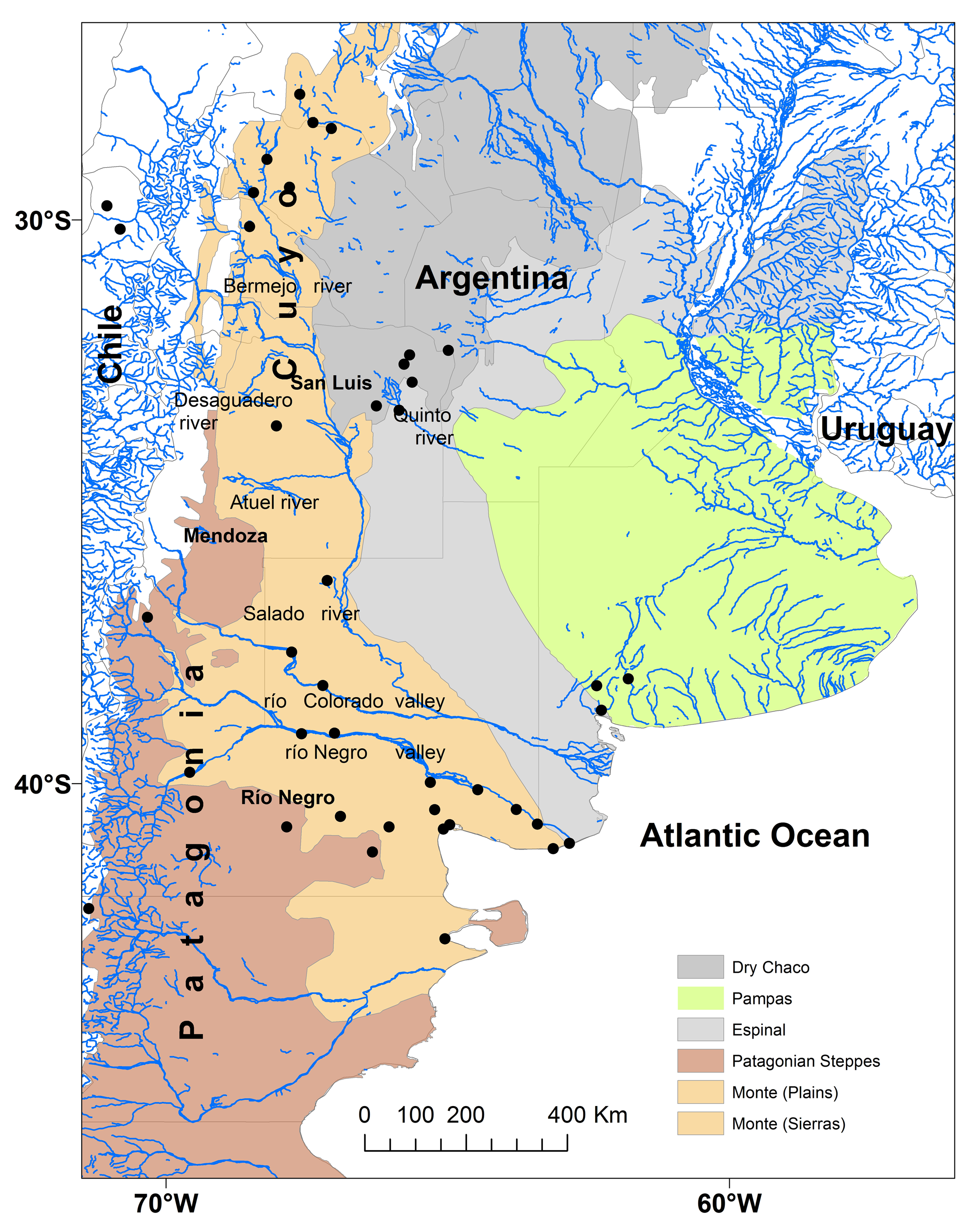

Supplement: Additional file 4 — Figure S1. Sample locations (black dots), main places, regions, and ecoregions mentioned in the text, and rivers of Southern South America. [file 1742-9994-8-16-S4.TIFF]
